# Rickets

**Published:** 1906-12-15

**Authors:** 


### SPECIAL ARTICLE.

### RICKETS.

Although so common a disease, rickets presents several points of mystery, particularly as regards the precise nature of the morbid process at work in the production of the lesions characteristic of the disease. It is, of course, certain that some widespread nutritional disturbance underlies the whole malady, and that the dietary which most often results in the production of rickets is one containing either an excess of carbo-hydrate elements, or a deficiency of fat, or, as commonly happens, both these disabilities. Young animals in captivity are quite often affected by rickets, a fact which dissipates the idea sometimes entertained that inheritance is a determining factor in the incidence of the malady. Experiments carried out by Bland-Sutton at the Zoological Gardens tend to prove that of all the elements of dietary, fat is that which is most closely associated with the production or avoidance of rickety manifestations. Thus in the case of lion-whelps weaned early and fed upon raw meat the appearance of rickety lesions is invariable. The same result attended the feeding of young monkeys upon a diet exclusively vegetable, while the influence of the administration of fats in dispersing the lesion of rickets is a commonplace of therapeutics.

We may assume, then, that rickets as we meet it in the case of infants is dependent upon a deficiency of fat in the diet or upon an excess of carbo-hydrate, or both; but even so, account must be taken of individual idiosyncrasy. Thus it is no rarity to observe that of two infants in the same family who have been fed upon a similar diet (let us say condensed milk) one will pass through a healthy infancy, while the other is profoundly rickety. Further, there is little doubt that in some instances mere excess of carbo-hydrate will produce rickets although the diet may have included an abundance of fat. For example, a boy of eighteen months was found to be extremely rickety although it was credibly reported of him by his mother, a sensible woman in a good position, that his daily menu habitually included two pints of cow's milk, obtained from an excellent source and administered either plain or in the shape of milk-pudding—such as rice-pudding. This child, although tolerably well-nourished, showed definite epiphysial lesions, and had not cut a single tooth.

The most obvious lesion of rickets is the well-know affection of the osseous system. Histologically this affection includes an excessive production of cartilage at the growing areas of long bones together with a deficiency of ossification, that is to say, a shortage in the deposit of calcareous salts in the cartilage so formed. Although the changes of rickets are most in evidence at the epiphysial regions of the long bones, a process of a similar nature commonly attacks the flat bones of the skull, with the production of bosses in the neighbourhood of the centres of ossification. These anomalies in development lead to the skeletal deformities which stamp the malady. Thus the cartilaginous overgrowth of the long bones produces nodular thickenings at their epiphyses, thickenings which are most obvious at the superficial epiphyses of the wrists and ankles, while a similar affection of the chondro-costal epiphyses results in the formation of the "rickety rosary." The bossing of the flat bones of the skull also contributes to the classical picture of the malady by producing a typical prominence of the forehead and of the parietal eminences, while retarded ossification leads to a prolonged patency of the anterior fontanelle.

In addition to these directly developmental deformities there occur a variety of other changes in bony outline indirectly produced, and attributable to the associated softness and flexibility of the ill-ossified bones. Of these the commonest are curvatures of the long bones, produced partly by the weight of the body and partly by muscular action. In consequence the legs are more frequently affected than the arms, and also to a greater extent. The typical curvature of the tibia is one appearing in the lower third of the bone, the convexity of the curve being directed forward and outward. This deformity is the basis of most rickety bow-legs. The deformity of the femur usually consists in a general curve forwards. This may be associated with an overgrowth of the internal condyle, which, assisted by the laxity of the ligaments of the knee-joint, results in the production of knock-knee. The radius and ulna are often affected, the curve in this case being convex towards the extensor aspect.

The shape of the thorax is often profoundly modified by the softness of its bony wall. This deformity is produced by the act of inspiration. The inward pressure upon the thorax exercised at each contraction of the intercostal muscles and diaphragm proves too great for the resistance of the chest-wall, which yields in consequence at its weakest point, namely the line of junction of the ribs with their cartilages. In this way is produced the transverse furrow at the level of the xiphoid cartilage to which the name of "Harrison's sulcus" is usually applied. Although this furrow is often limited to the lower part of the chest, it may extend upwards parallel with the sternum and produce a variety of pigeon breast. In a majority of cases rachitic deformities of the chest do not depend entirely upon the softness of the thoracic skeleton, but to a large extent upon contributory lesions of the respiratory passages commonly associated with the constitutional condition of the rickety, lesions to be considered in further detail below.

Now, although the bone lesions of rickets acquire a dramatic prominence which is indifferently shared by the general constitutional disturbances induced by this disease, it is to the latter that the condition owes the greater part of its potential gravity. The nutritional defects so obviously manifested by the osseous system are reflected by every other system in the body. The muscular system shows its disabilities in a weakness and flabbiness of the muscles, which, associated with laxity of the ligaments of the joints, leads sometimes to a curvature of the spine, a kyphosis in the lower dorsal and lumbar regions, and always to a late assumption of the erect position. The rickety child is backward in every department of progress, but in none more noticeably so than in the power to walk. The alimentary system is profoundly affected. Dentition is habitually delayed, to such an extent sometimes that the second year may be completed without the eruption of a single tooth. At the same time there appears physical evidence of gastro-intestinal trouble. The belly becomes big and tympanitic, while the motions of the bowels evince a marked irregularity, periods of diarrhœa alternating with periods of constipation. This abdominal distension is constant in the established disease. It depends in part upon the flatulent distension of the intestines by the gaseous products of abnormal fermentation, in part upon the enlargement of the liver and spleen which usually marks the disease, and in part, probably, upon the tonelessness of the muscles of the anterior abdominal wall. The lesions of the respiratory system often include the development of adenoid vegetations in the pharynx and naso-pharynx, to which, in conjunction with the naso-pharyngeal catarrh thereby set up, may be traced the great liability to bronchitic affections evinced by rickety children. Further, the obstacle to the free ingress of air into the thorax provided by such pharyngeal growths and their sequelæ in the lungs, supplies an increased tax upon the weakened thoracic walls and aggravates the deformities of the chest to which we have referred above. As a natural consequence of all these physical influences we find in rickety children a great tendency towards pulmonary collapse.

The nervous system in this malady shows many evidences of irritability and unstableness. The sleep of rickety infants is habitually broken, while their waking hours are marked by a fretfulness which shows clearly enough that the body is ill at ease. Profuse sweats during sleep are a pronounced feature of the rickety constitution, and contribute towards the frequency of "colds" in such subjects. The general irritability of the nervous system shows itself fairly often in certain well-defined explosions of nervous force. Of these the commonest is represented by general convulsions induced by stimuli intrinsically trifling, such, for instance, as some slight gastro-intestinal irregularity, or the irritation associated with the eruption of teeth. The recurrent spasm of the glottis, which goes by the name of "laryngismus stridulus," is seldom met with except in rickety infants. This is a most alarming affection in some instances, and occasionally, though rarely, proves fatal. It is characterised by a sudden dyspnœa, associated with cyanosis and obvious manifestations of terror on the part of the child. The respiratory obstruction appears to be absolute while the spasm lasts, and, with the increasing cyanosis and distress of the victim, gives the impression that some urgent interference is demanded, when, after an interval of some fifteen or twenty seconds, the spasm yields and inspiration is once more effected to the accompaniment of a loud crowing sound. Yet another nervous explosion in the rachitic presents itself in the condition known as tetany or carpo-pedal spasm. Although this condition is not confined to children, yet when it occurs in early life it is almost invariably among subjects of the general disorder with which we have to do. Tetany is a symmetrical tonic muscular spasm. The joints most frequently affected are the wrists and ankles and the parts below them. The attitude typical of the disease includes a flexion of the wrist with adduction and flexion of the thumb upon the palm. The fingers are strongly flexed at the meta-carpo-phalangeal joint, while the inter-phalangeal joints are strongly extended. The affection of the ankle is similar. Thus, the feet assume the position of extreme equinus, while the toes behave in a fashion similar to the fingers. The condition is accompanied by a great irritability of nerve-trunks, which has led to the description of two symptoms held to be characteristic. Of these one is called "Chvostek's symptom," and consists in a contraction of muscle when the nerve-trunk supplying it is smartly struck. This symptom is most easily elicited by percussion of the facial nerve in front of the ear. A similar result may follow compression of a nerve-trunk, in which case the reaction is known as "Trousseau's symptom."

The diagnosis of rickets in a well-marked case hardly admits of doubt. The age of the patient and the bone lesions typical of the disease are generally conclusive. But doubt occasionally occurs as to whether or not rickets alone is responsible for certain symptoms which are shared by other diseases. For instance, an opinion may be required as to whether general backwardness in a rickety child is to be credited to rickets alone or to some associated mental deficiency, whether primarily cerebral or secondary to such a state as cretinism. This distinction may offer great difficulty. The points upon which reliance should be placed are these: Idiocy and cretinism are both as a rule manifest earlier in life than the age at which rickets commonly makes its appearance, for rickets is seldom seen earlier than the sixth month. Therefore, if a backward and rickety child presented the appearance of normal health for the first six months of life it is probable that rickets is the sole responsible cause of the backwardness. Sometimes the extreme muscular feebleness of rickets may give rise to suspicion of paralysis; but in such a case careful search for bony changes and a consideration of the electrical reactions and reflexes will usually suffice to effect a distinction. The most prominent symptoms of the early disease are the delay in the closure of the anterior fontanelle and the copious sweating of the skin during sleep.

The prognosis of rickets turns largely upon the secondary accidents of the condition, for the disease itself is never fatal. But there is no doubt that the general debility associated with the constitutional condition paves the way for the assault of many diseases which bulk largely in the mortality-bills of early childhood. Of these the most conspicuous are broncho-pneumonia and gastro-enteritis. Very considerable degrees of bony deformity may disappear without calling for surgical interference, but rickets of any severity generally results in some stunting of growth. The rational treatment of rickets is made sufficiently plain by a consideration of its ætiology. It is purely a matter of qualitative dietetics. It is well at the beginning of treatment to put an absolute embargo upon carbohydrates, excepting the milk-sugar occurring naturally in milk, and to see that fat is as copiously represented in the diet as the digestive capacity of the patient permits.

